# Friedreich Ataxia Patient Tissues Exhibit Increased 5-Hydroxymethylcytosine Modification and Decreased CTCF Binding at the *FXN* Locus

**DOI:** 10.1371/journal.pone.0074956

**Published:** 2013-09-04

**Authors:** Sahar Al-Mahdawi, Chiranjeevi Sandi, Ricardo Mouro Pinto, Mark A. Pook

**Affiliations:** 1 Ataxia Research Group, Division of Biosciences, School of Health Sciences and Social Care, Brunel University, Uxbridge, Middlesex, United Kingdom; 2 Molecular Neurogenetics Unit, Center for Human Genetic Research, Massachusetts General Hospital, Boston, Massachusetts, United States of America; National Institute for Medical Research, Medical Research Council, London, United Kingdom

## Abstract

**Background:**

Friedreich ataxia (FRDA) is caused by a homozygous GAA repeat expansion mutation within intron 1 of the *FXN* gene, which induces epigenetic changes and *FXN* gene silencing. Bisulfite sequencing studies have identified 5-methylcytosine (5mC) DNA methylation as one of the epigenetic changes that may be involved in this process. However, analysis of samples by bisulfite sequencing is a time-consuming procedure. In addition, it has recently been shown that 5-hydroxymethylcytosine (5hmC) is also present in mammalian DNA, and bisulfite sequencing cannot distinguish between 5hmC and 5mC.

**Methodology/Principal Findings:**

We have developed specific MethylScreen restriction enzyme digestion and qPCR-based protocols to more rapidly quantify DNA methylation at four CpG sites in the *FXN* upstream GAA region. Increased DNA methylation was confirmed at all four CpG sites in both FRDA cerebellum and heart tissues. We have also analysed the DNA methylation status in FRDA cerebellum and heart tissues using an approach that enables distinction between 5hmC and 5mC. Our analysis reveals that the majority of DNA methylation in both FRDA and unaffected tissues actually comprises 5hmC rather than 5mC. We have also identified decreased occupancy of the chromatin insulator protein CTCF (CCCTC-binding factor) at the *FXN* 5’ UTR region in the same FRDA cerebellum tissues.

**Conclusions/Significance:**

Increased DNA methylation at the *FXN* upstream GAA region, primarily 5hmC rather than 5mC, and decreased CTCF occupancy at the *FXN* 5’ UTR are associated with FRDA disease-relevant human tissues. The role of such molecular mechanisms in FRDA pathogenesis has now to be determined.

## Introduction

FRDA is an autosomal recessive neurodegenerative mitochondrial disorder caused primarily by a homozygous GAA repeat expansion mutation within intron 1 of the *FXN* gene [1]. Unaffected individuals have up to 43 GAA repeats, while affected individuals have 44 to 1700 GAA repeats, most commonly between 600–900 GAA repeats [2,3]. The length of the smaller GAA repeat correlates with FRDA disease severity and inversely correlates with the age of onset [4,5]. The effect of the GAA repeat expansion is to decrease expression of the essential and ubiquitously expressed mitochondrial frataxin protein, with levels in FRDA patients ranging from 4% to 29% that of normal [6]. Reduced levels of frataxin in FRDA patients are associated with defects of iron–sulphur (Fe–S) cluster biosynthesis [7], mitochondrial iron accumulation in the heart, spinal cord and dentate nucleus of the cerebellum [8] and increased susceptibility to oxidative stress [9].

Two main hypotheses have been proposed to link *FXN* GAA repeat expansions with decreased frataxin expression. Firstly, evidence from *in vitro* and cell transfection studies suggests that GAA repeat expansions may adopt abnormal non-B DNA structures (triplexes or “sticky DNA”) or DNA•RNA hybrid structures (R-loops), which impede the process of RNA polymerase II and thus reduce *FXN* gene transcription [10,11]. Secondly, there is evidence that GAA repeat expansions can induce heterochromatin-mediated gene silencing effects [12]. Consistent with the latter hypothesis, several FRDA-related epigenetic changes have been identified in the immediate vicinity of the expanded GAA repeats of the *FXN* gene [13]. An initial investigation of DNA methylation of the *FXN* gene by bisulfite sequencing revealed hypermethylation of the cytosine residue of specific CpG sites upstream of the GAA repeat sequence in FRDA patient lymphoblastoid cells compared to cells derived from unaffected individuals [14]. We have subsequently used bisulfite sequencing to identify increased DNA methylation at the upstream GAA repeat region in FRDA patient cerebellum and heart autopsy tissues, which are clinically relevant tissues in FRDA [15]. Interestingly, we also identified reduced levels of DNA methylation in the downstream GAA repeat region in FRDA patient tissues compared with controls. These findings have now been confirmed by bisulfite-based EpiTYPER MassARRAY analysis of blood and buccal cell samples from a large cohort of FRDA patients, where a significant inverse correlation was also detected between the level of DNA methylation in the upstream GAA region and the level of *FXN* expression [16]. Yet another study has shown that the degree of DNA methylation in the upstream GAA repeat region in FRDA patients correlates with the length of the GAA repeats and inversely correlates with the age of disease onset [17]. Therefore, there is good evidence that DNA methylation may have some role to play in the molecular mechanism of GAA repeat induced FRDA disease. However, bisulfite sequencing is a somewhat time-consuming procedure. Therefore, we now report a more rapid MethylScreen restriction enzyme digestion and qPCR procedure, which we have validated by confirming increased DNA methylation at the upstream GAA repeat region in FRDA patient cerebellum and heart autopsy tissues.

Methylation of mammalian DNA primarily occurs as 5mC modification of CpG dinucleotides. However, recent studies have revealed the existence of an alternative modification, 5hmC, which is formed by oxidation of 5mC by ten-eleven translocation (TET) enzymes [18,19]. The overall levels of 5hmC in the mammalian genome are approximately 10% of 5mC levels [20], although higher levels have been detected in tissues of the central nervous system [21]. For example, 5hmC is approximately 40% as abundant as 5mC in the DNA of Purkinje cells of the cerebellum [18]. There are two main theories regarding the function of 5hmC. Firstly, 5hmC may be an intermediate in the removal of 5mC by an active demethylation process that involves the base excision repair pathway [22]. Secondly, 5hmC may be an epigenetic modification in its own right, regulating chromatin or transcriptional factors involved in processes such as neurodevelopment [23]. Therefore, it is possible that 5hmC may also be involved in epigenetic-based disease mechanisms, such as those proposed for FRDA. However, useful information cannot be obtained from previous bisulfite sequence DNA methylation data, because this method does not distinguish between 5mC and 5hmC [24]. Therefore, we have analysed the DNA methylation status of one of the *FXN* upstream GAA CpG sites in FRDA cerebellum and heart tissues using a procedure that does allow distinction between 5hmC and 5mC and we report that the majority of the methylated DNA at this CpG residue comprises 5hmC rather than 5mC.

In addition, another factor that has been proposed to be involved in silencing of the *FXN* gene in FRDA is the chromatin insulator protein CTCF, because decreased occupancy of CTCF has been identified at the *FXN* 5’ UTR region of FRDA fibroblast cells [25]. Therefore, we have decided to further investigate CTCF occupancy in FRDA cerebellum tissue and we report decreased occupancy of CTCF at the *FXN* 5’ UTR region. Overall we show that increased 5hmC at the *FXN* upstream GAA region and decreased CTCF occupancy at the *FXN* 5’ UTR are both associated with FRDA disease-relevant human tissues.

## Materials and Methods

### DNA preparation from human tissues

Human cerebellum and heart tissue samples were obtained from autopsies of four FRDA patients (aged 17, 24, 36 and 47, each homozygous for GAA repeat expansions within the range of approximately 550-750 units) and four unaffected individuals (aged 69-82, obtained from NDRI). Research ethical approval was obtained from the SHSSC Research Ethics Committee, Brunel University, and samples were obtained with written consent and were stored in accordance with UK Human Tissue Authority ethical guidelines. For the FRDA patient aged 17, written informed consent was obtained from the next of kin under the organisation of the Friedreich’s Ataxia Group tissue donor scheme. Genomic DNA was isolated from frozen tissues by standard phenol/chloroform extraction and ethanol precipitation.

### MethylScreen analysis

**Figure 1:**
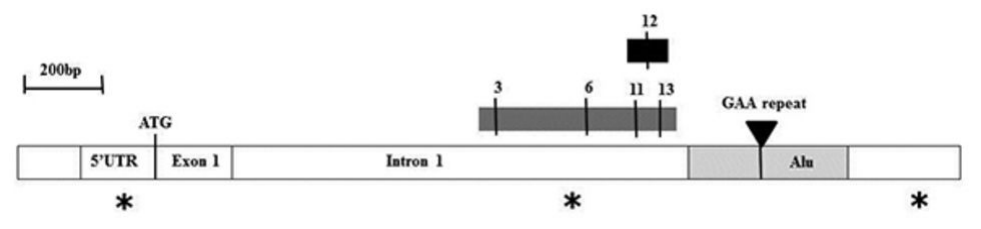
*FXN* region of analysis. Schematic diagram of 2.5kb of the *FXN* exon 1 and intron 1 region showing the GAA repeat within an Alu sequence (light grey box), together with the regions of 5mC/5hmC MethylScreen (dark grey box) and 5hmC EpiMarkTM (black box) analysis. The numbers represent the CpG sites, which are numbered according to [13]. Asterisks indicate the positions of the three potential CTCF binding sites that were analysed by EMSA.

Four CpG sites in the *FXN* upstream GAA repeat region, designated 3, 6, 11 and 13 (numbered according to [14], Figure 1), were investigated using adaptations of the previously described MethylScreen assay [26]. 1µg of genomic DNA was digested with: (1) a methylation-sensitive restriction enzyme (MSRE), (2) a methylation-dependent restriction enzyme (MDRE), (3) both MSRE and MDRE (double digest, DD), and (4) neither MSRE or MDRE (mock control). The MSREs used for CpGs 3, 6, 11 and 13 were *Aci*I (Fermentas), *Hpy*188III (New England Biolabs), *Aji*I (Fermentas) and *Eco*72I (Fermentas), respectively. The MDRE used for all four CpGs was *Mcr*Bc (Fermentas). A 50ng aliquot of digested DNA was then amplified by quantitative PCR using SYBR^®^ Green (Applied Biosystems) and an ABI7900HT Fast Real-Time PCR System with the following primers: CpG3 F 5’-GAGACGTGGCTTTGTTTTCTG-3’ and R 5’-GTTTCCTCCTTTCAAGCCGTG-3’; CpG6 F 5’-GAAGATGCCAAGGAAGTGGTAG-3’ and R 5’-GAGCAACACAAATATGGCTTGG-3’; CpG11 and CpG13 F-met 5’-GAACCGTCTGGGCAAAGGCCAG-3’ and R-met 5’-ATCCCAAAGTTTCTTCAAACACAATG-3’. PCR quantification was carried out using the ΔCt method (values were calculated as 2 ^ΔCt (mock – digest)^ with the mock value set at 100%) and RQ Manager software (Applied Biosystems). Each qPCR reaction for each sample was performed in triplicate in a 96-well plate. MethylScreen DNA methylation values were then calculated as follows: Densely methylated (DM) = (MSRE-DD)/(100-DD) x 100; unmethylated (UM) = (MDRE-DD)/(100-DD) x 100; intermediately methylated (IM) = 100 - (DM+UM).

### 5hmC analysis

Levels of 5mC and 5hmC were determined at CpG site 12 (numbered according to [14]) of the *FXN* upstream GAA repeat region (Figure 1) using the EpiMark^TM^ Analysis Kit (New England Biolabs). 1µg genomic DNA was glucosylated with T4 B-glucosyltransferase (T4-BGT), and then both glucosylated (G) and non-glucosylated (NG) DNA samples were digested with the methylation sensitive restriction enzyme, *Hpa*II, or the methylation insensitive restriction enzyme, *Msp*I. Digested DNA, together with non-digested (mock control) DNA samples were then amplified by quantitative PCR using SYBR^®^ Green (Applied Biosystems) on an ABI7900HT Fast Real-Time PCR System with F-met and R-met PCR primers (the same as used for MethylScreen analysis). PCR quantification was carried out using the ΔCt method (values were calculated as 2 ^ΔCt (mock+G – digest)^ with the mock+G value set at 100%) and RQ Manager software (Applied Biosystems). Each qPCR reaction for each sample was performed in duplicate in a 96-well plate.

### CTCF electrophoretic mobility shift assay (EMSA)

EMSA was performed with recombinant CTCF protein and three γ-^32^P end-labelled *FXN* probes, together with CTCF-positive (DM1 (1)) and CTCF-negative (DM1 (3)) probes [27]. The probes were prepared by PCR amplification of human genomic DNA using the following primer pairs: *FXN* 5’ UTR, F 5’-AAGCAGGCTCTCCATTTTTG-3’, R 5’-CGAGAGTCCACATGCTGCT-3’; *FXN* GAA Upstream, F 5’-GAAACCCAAAGAATGGCTGTG-3’, R 5’-TTCCCTCCTCGTGAAACACC-3’; *FXN* GAA Downstream, F 5’-TGGGTTGTCAGCAGAGTTGT-3’, R 5’-CCGATAATCCCAGCTACTCG-3’; DM1 (1), F 5’-GCCTGCCAGTTCACAACC-3’, R 5’-AGCAGCATTCCCGGCTAC-3’; DM1 (3), F 5’-AGCTTTCTTGTGCATGACG-3’, R 5’-GGTTGTTGGGGGTCCTGTAG-3’. PCR products were gel purified using Geneclean III (BIO 101) and end-labelled with γ-^32^P ATP using T4 polynucleotide kinase. Labelled PCR products were put through a Microspin S-200 HR column (GE HealthCare) and then incubated with 0.2µg of CTCF full-length recombinant protein (Abnova) in PBS buffer containing 5mM MgCl_2_, 0.1mM ZnSO_4_, 1mM DTT, 0.1% Nonidet P-40, 10% glycerol and 1µg of double-strand competitor DNA poly(dI-dC) (Sigma). After incubation for 30 minutes at room temperature, samples were run on a 5% nondenaturing polyacrylamide gel in 0.5X TBE buffer at 100V for 1-2 hours. Gels were then vacuum dried and exposed to ECL Hyperfilm (GE HealthCare), followed by film development.

### CTCF chromatin immunoprecipitation (ChIP)

Human cerebellum tissue samples (30mg from each of three FRDA and three unaffected controls, the same as used for DNA preparation) were homogenised, followed by cross-linking of DNA and protein by formaldehyde treatment. Cell nuclei were isolated by centrifugation and then lysed in the presence of protease inhibitors [15]. DNA was sheared by sonication, followed by immunoprecipitation with anti-CTCF antibody (Upstate 07-729). After reversal of cross-linking, qPCR amplification of the resultant co-immunoprecipitated DNA was carried out with SYBR^®^ Green in an ABI7400 sequencer (Applied Biosystems) using *FXN* 5’ UTR primers and *GAPDH* (negative for CTCF) control primers as previously described [25]. PCR quantification was carried out using the ΔΔCt method and RQ Manager software (Applied Biosystems). Each value of immunoprecipitated DNA was normalised to both input and *GAPDH* values. Each tissue sample was subjected to four independent ChIP procedures, followed by triplicate qPCR analysis.

### Statistical analysis

Comparison of data from FRDA patients and unaffected control samples was carried out using the student’s t test with a significance value set at *P*<0.05.

## Results

### MethylScreen analysis is a rapid method to quantify DNA methylation in the *FXN* upstream GAA repeat region

**Figure 2:**
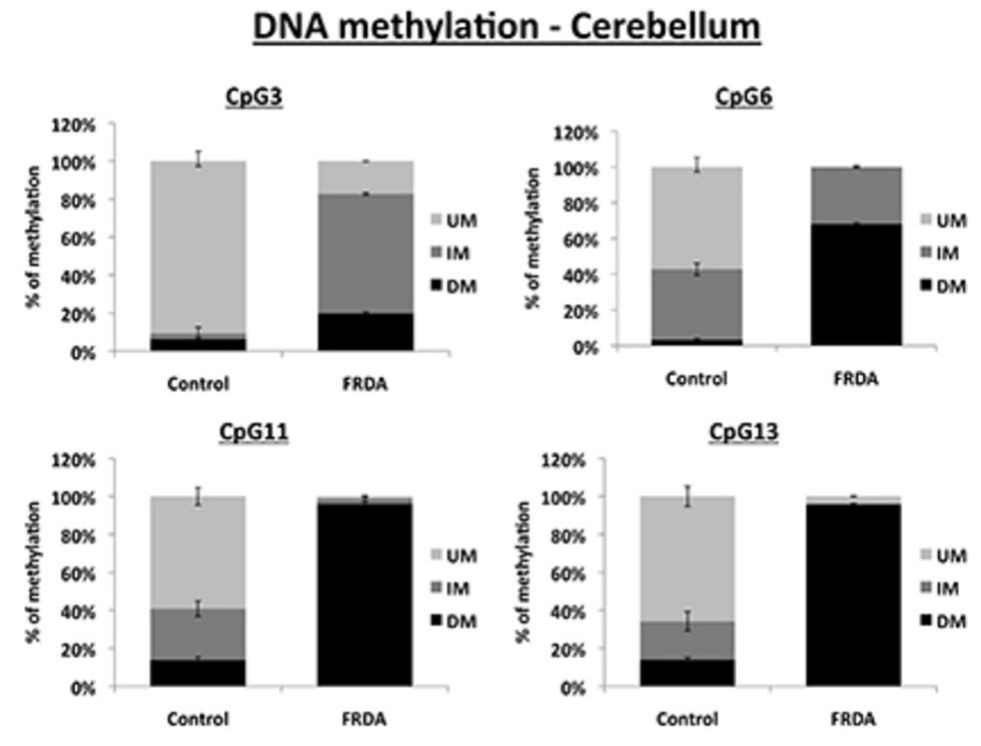
DNA methylation levels in cerebellum. MethylScreen analysis of four CpG sites in the FXN upstream GAA repeat region of DNA from FRDA and control cerebellum tissues. UM = unmethylated, IM = intermediately methylated, DM = densely methylated. Error bars = s.e.m. n=4.

**Figure 3:**
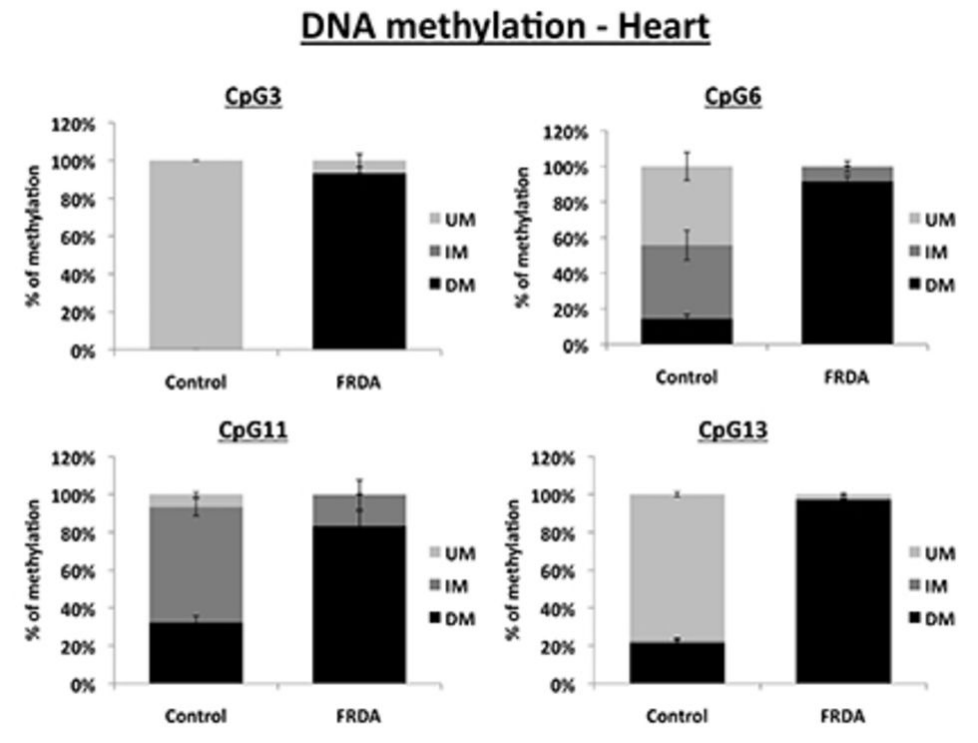
DNA methylation levels in heart. MethylScreen analysis of four CpG sites in the FXN upstream GAA repeat region of DNA from FRDA and control heart tissues. UM = unmethylated, IM = intermediately methylated, DM = densely methylated. Error bars = s.e.m. n=4.

We, and others, have previously used bisulfite sequencing to quantify DNA methylation changes at the *FXN* upstream GAA repeat region in FRDA cells and tissues [14–17]. However, bisulfite sequencing is a somewhat time-consuming procedure, which can be subject to errors due to incomplete bisulfite treatment. Therefore, we have now adapted the previously described MethylScreen assay [26] to rapidly quantify the DNA methylation status at four CpG sites at the *FXN* upstream GAA repeat region, designated 3, 6, 11 and 13 according to [14] (Figure 1). The MethylScreen method does not require bisulfite treatment of DNA, but involves digestion of genomic DNA with a MSRE, a MDRE, and both a MSRE and a MDRE, followed by qPCR amplification of the residual non-digested DNA using an amplicon that spans the CpG site of interrogation. The output data is expressed as three levels of DNA methylation status: unmethylated (UM), intermediately methylated (IM) and densely methylated (DM). We validated the MethylScreen assay by investigating the levels of DNA methylation at CpG11 and CpG 13 in cerebellum and heart tissue from FRDA patients and unaffected controls, which allowed a direct comparison with our previously reported bisulfite sequence analysis [15]. In addition, we investigated CpG3 and CpG6 in cerebellum and heart tissue, since these sites together with CpG13 have previously been identified as the three sites of most significant DNA methylation increase in FRDA versus control lymphoblastoid cells [14]. Our results for cerebellum tissue reveal an increase in DNA methylation at all four CpG sites in FRDA tissue compared with unaffected control tissue, in agreement with all previous bisulfite sequencing data (Figure 2). However, the magnitude of this DNA methylation increase was found to vary from a non-statistically significant small increase in DM of 6% to 20% at CpG3, through an intermediate level of increase in DM of 4% to 68% at CpG6 (*P*<0.001), to high levels of increase in DM of 14% to 96% at both CpG11 and CpG13 (both *P*<0.001). The results for heart tissue also reveal significant increases in DNA methylation at all four CpG sites in FRDA tissue compared with unaffected control tissue, with DM values increasing from 0% to 93% at CpG3 (*P*<0.001), 15% to 92% at CpG6 (*P*<0.001), 32% to 83% at CpG11 (*P*<0.01) and 21% to 97% at CpG13 (*P*<0.001), again in agreement with all previous bisulfite sequencing data (Figure 3). Therefore, the specific MethylScreen protocols that we have developed can be considered as both rapid and robust procedures to investigate DNA methylation status at the *FXN* locus.

### 5hmC is the predominant constituent of DNA methylation in the *FXN* upstream GAA repeat region and is increased in FRDA cerebellum and heart tissues

Levels of 5mC and 5hmC were determined at CpG site 12 of the *FXN* upstream GAA repeat region (Figure 1) using the EpiMark^TM^ Analysis Kit (New England Biolabs). This CpG site was chosen because it resides within a CCGG motif, which is required by the EpiMark^TM^ procedure for differential restriction enzyme digestion by *Hpa*II and *Msp*I. The principle of this technique depends on the addition of glucose to the hydroxyl group of 5hmC via an enzymatic reaction utilising T4-BGT. Genomic DNA is glucosylated, then digested with a methylation sensitive restriction enzyme, *Hpa*II, and a methylation insensitive restriction enzyme, *Msp*I. If 5hmC is present in the context of CCGG, this modification converts a normally cleavable *Msp*I site to a non-cleavable one.

**Figure 4:**
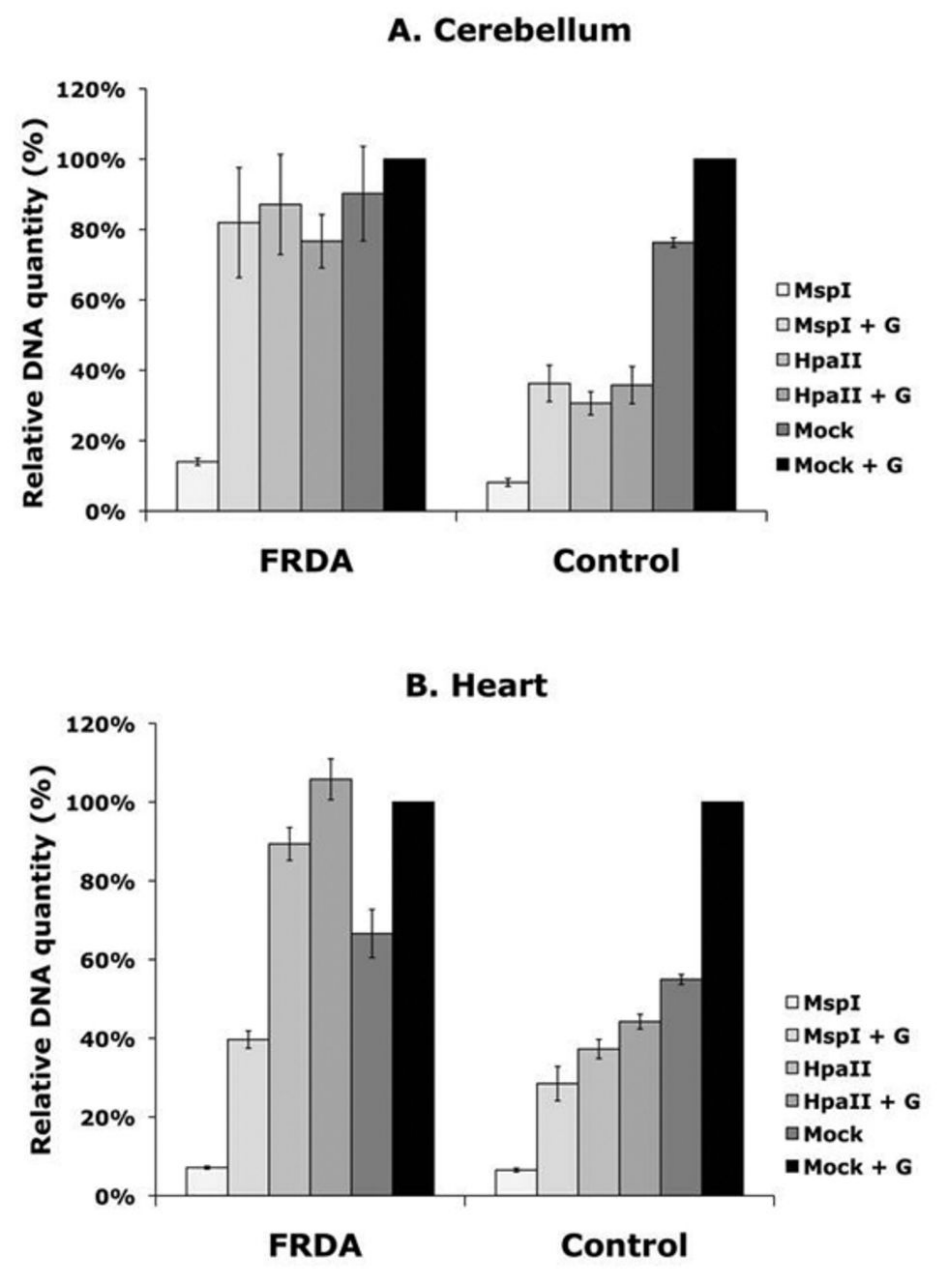
5hmC levels in cerebellum and heart. EpiMark^TM^ 5hmC and 5mC analysis of CpG site 12 in the FXN upstream GAA repeat region of DNA from FRDA and control cerebellum (**A**) and heart (**B**) tissues. G = glucosylated DNA. MspI values represent complete digestion control levels of DNA; MspI + G represents 5hmC levels, HpaII and HpaII + G represent combined 5mC and 5hmC levels; Mock and Mock +G represent undigested control levels of DNA. Error bars = s.e.m. n=4.

Initial determination of DNA methylation levels (combined 5mC and 5hmC) by observing *Hpa*II + G and *Hpa*II qPCR values at CpG12 in cerebellum tissue reveals a significant increase in DNA methylation in FRDA tissue compared with unaffected control tissue (Figure 4A), at levels similar to those previously identified by bisulfite sequencing. Thus, DNA methylation levels increase from either 36% to 77% (*Hpa*II + G, *P*<0.01) or 30% to 87% (*Hpa*II, *P*<0.05) compared with previously reported bisulfite sequence values of 40% to 95% [15]. However, more detailed analysis reveals that there is no difference in the *Msp*I + G and *Hpa*II + G qPCR results from unaffected cerebellum tissues, and similarly there is no difference in the *Msp*I + G and *Hpa*II + G qPCR results from FRDA cerebellum tissues (Figure 4A). This indicates that all methylated DNA in both the unaffected and the FRDA cerebellum tissue comprises 5hmC rather than 5mC. Therefore, we can now conclude that a characteristic feature of FRDA cerebellum tissue is an increase in 5hmC-modified DNA in the *FXN* upstream GAA repeat region, at least at CpG site 12. Determination of DNA methylation levels (combined 5mC and 5hmC) in heart tissue also reveals a significant increase in DNA methylation in FRDA tissue compared with unaffected control tissue (Figure 4B), at levels similar to those previously identified by bisulfite sequencing. Thus, DNA methylation levels increase from either 44% to 100% (*Hpa*II + G, *P*<0.01) or 37% to 89% (*Hpa*II, *P*<0.001) compared with previously reported bisulfite sequence values of 65% to 95% [15]. However, more detailed analysis of the *Msp*I + G compared with *Hpa*II + G qPCR results indicates that the methylated DNA comprises approximately 64% 5hmC in the unaffected heart tissues and only 37% 5hmC in the FRDA heart tissues, although there is still an overall increase in 5hmC levels (*Msp*I + G values) in FRDA compared with controls (Figure 4B). This suggests that, while there is increased DNA methylation in both cerebellum and heart tissues, there are also tissue specific differences: DNA methylation is entirely due to 5hmC in the cerebellum, but only partially due to 5hmC in the heart.

### CTCF occupancy at the *FXN* 5’ UTR region is decreased in FRDA cerebellum tissue

**Figure 5:**
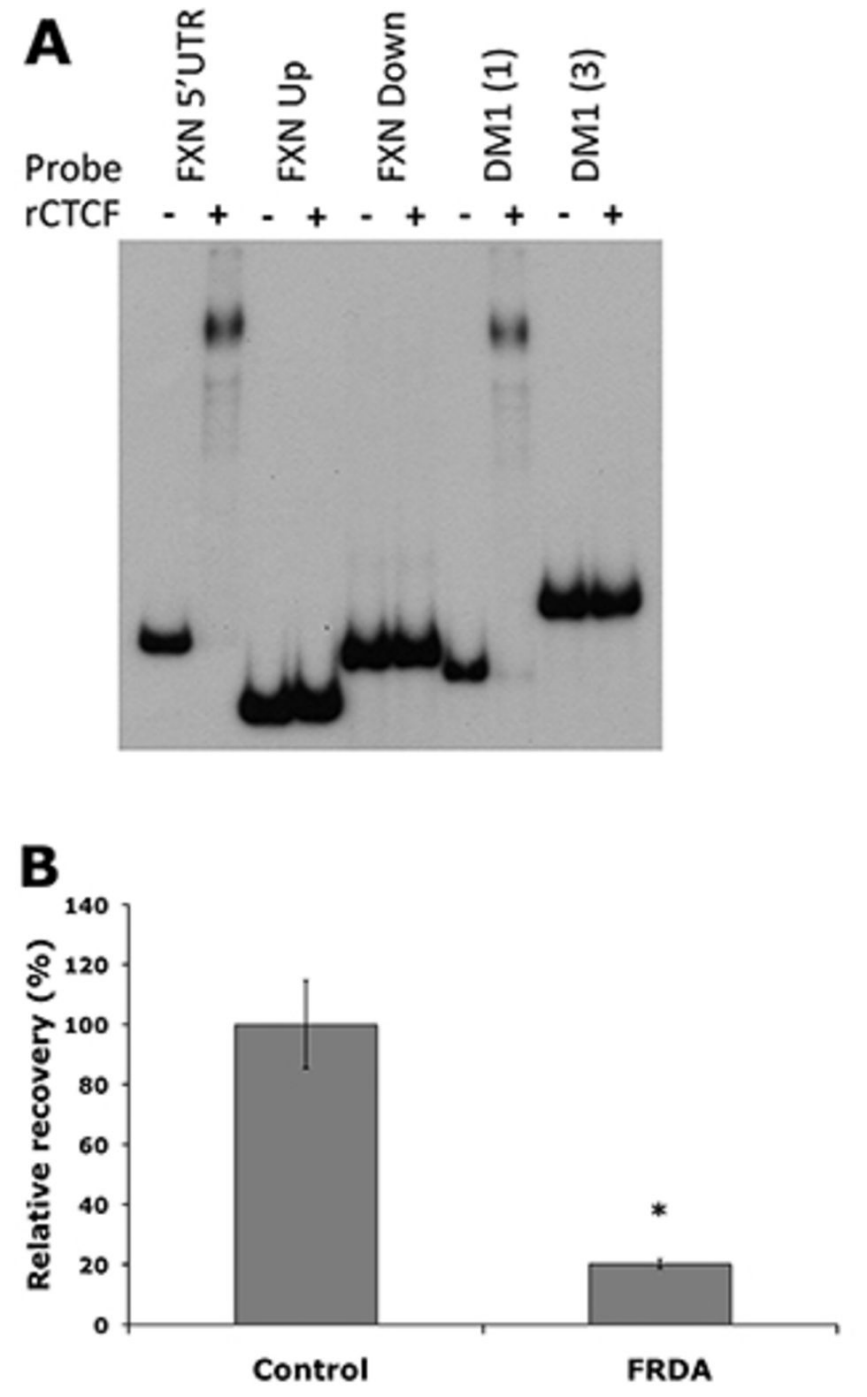
CTCF analysis. (**A**) EMSA showing a mobility shift for the *FXN* 5’ UTR probe and CTCF-positive DM1 (1) probe [35], while there is no such shift detected for the FXN upstream GAA (Up) probe, *FXN* downstream GAA (Down) probe or CTCF-negative DM1 (3) probe [35]. (**B**) ChIP analysis showing the relative CTCF occupancy in the *FXN* 5’ UTR of DNA from FRDA and control cerebellum tissues. Error bars = s.e.m. n=3. * = P<0.05.

We firstly performed *in silico* screening of a 40kb region spanning the *FXN* gene for potential CTCF binding sites using the bioinformatics web tool designed by Klenova and colleagues (http://www.essex.ac.uk/bs/molonc/spa.htm) and we identified three regions of interest at the start of the *FXN* gene. We then investigated the three potential CTCF binding regions, which were located within the 5’ UTR, GAA upstream and GAA downstream regions of the *FXN* locus (Fig 1), by EMSA using recombinant full length CTCF protein. Only the 5’ UTR probe proved capable of binding CTCF protein to cause a gel shift (Figure 5A). This result is in agreement with a previous report that identified CTCF binding in the *FXN* 5’ UTR region using fibroblast cell lysates [25]. We then performed ChIP analysis to assess the level of CTCF binding at the 5’ UTR site in three FRDA cerebellum tissue lysates compared with three unaffected controls, using the same qPCR amplicons as previously described [25]. A statistically significant decrease (*P*<0.05) in CTCF binding was detected in FRDA cerebellum, with levels falling to only 20% of the levels detected in unaffected cerebellum controls (Figure 5B).

## Discussion

We have investigated two molecular mechanisms that may be involved in the disease process in FRDA patient cerebellum and heart tissues. Firstly, we have developed a rapid and robust MethylScreen method to determine the general DNA methylation status (5mC and 5hmC) at the *FXN* upstream GAA repeat region. The level of DNA methylation in FRDA cerebellum tissue showed a gradation across the four CpG sites investigated, progressively increasing as you move closer to the GAA repeat locus. This suggests that the GAA repeat expansion mutation may act as focal point to induce only a localised effect of DNA methylation, spreading upstream from this point. However, this gradation effect was not seen in FRDA heart tissue, which demonstrated high levels of DNA methylation at all 4 CpG sites, indicating a degree of variability in the DNA methylation process in different FRDA tissues. Such variability may have some bearing on the previously identified differences in GAA repeat somatic instability, with cerebellum showing high levels of instability, while heart is comparatively stable [28].

Subsequently, we have shown that the methylated DNA at one CpG site in the *FXN* upstream GAA region comprises entirely 5hmC in FRDA cerebellum, but only partially 5hmC in FRDA heart. Our results are in agreement with the recent report that 5hmC is particularly associated with human cerebellum throughout development [29]. However, we can discount the possibility that the increased 5hmC that we have observed in FRDA tissues is a non-specific age-related effect in neuronal tissues, as previously reported for mouse hippocampus and cerebellum [23,30], because our FRDA patients are actually younger than the unaffected controls. Further studies will be required to determine if our finding is a general increased 5hmC effect for CpG sites within the *FXN* upstream GAA region, or indeed within other regions of the genome. The high levels of 5hmC that we have detected at the *FXN* upstream GAA region could be explained by either a passive or an active process. Thus, 5hmC could merely be a non-functioning intermediate in a demethylation process that is attempting to reverse the GAA repeat expansion-induced 5mC DNA methylation that is associated with *FXN* gene silencing [17]. In this case, the differences that we have detected between 5hmC levels in cerebellum and heart tissues could perhaps be due to tissue-selective differences in the efficiency of the TET enzymes that convert 5mC to 5hmC [18,19] or the DNA repair systems (e.g. base excision repair) that are required to convert 5hmC to cytosine [22]. Alternatively, 5hmC may have an as yet unidentified role in regulation of gene expression. For example, it has shown *in vitro* that 5hmC at the promoter but not in the gene body negatively regulates gene expression [31]. Furthermore, it has been proposed that 5hmC may be involved in the regulation of genes that are silent but transcriptionally poised for activation, where there is loss of 5mC, recruitment of Polycomb repressive complex 2 (PRC2) and, therefore, methylation of histone 3 at lysine 27 (H3K27me3) [20]. Indeed, 5hmC and H3K27me3 have been shown to have high correlation across a variety of somatic tissues [32]. Therefore, it is interesting to note that the *FXN* gene demonstrates RNA polymerase II pausing and FRDA lymphoblastoid cells have shown increased levels of H3K27me3 in the *FXN* upstream GAA repeat region [33,34]. In addition, it has been proposed that 5hmC may inhibit DNMT1 binding and may therefore function as a negative regulator of 5mC levels at protein-DNA interaction sites, allowing the binding of enhancer proteins or transcription factors [35]. Furthermore, 5hmC has been reported to bind MECP2, particularly in the CNS, although the functional consequences of such an interaction are not yet known [36]. Although the close positioning of the GAA repeat expansion mutation and the increased 5hmC modification of DNA in the *FXN* upstream GAA repeat region in FRDA would suggest a likely causal relationship, there may also be other more general factors involved in the formation of 5hmC. For example, the TET enzymes that produce 5hmC are known to be dependent on Fe(II) and α-ketoglutarate. Therefore, disruptions of iron metabolism, oxidative stress and the TCA cycle, processes which are highly relevant to FRDA, may all potentially affect 5hmC levels [37].

The second molecular change that we have identified in FRDA cerebellum is reduced CTCF occupancy at the *FXN* 5’ UTR region, confirming previous findings in FRDA fibroblast cells [25]. It has been hypothesized that decreased binding of CTCF in FRDA cells may be an epigenetic switch that allows increased frataxin antisense (*FAST-1*) transcription, spread of heterochromatin and resultant *FXN* gene silencing. DNA methylation has not been thought to play an active role in this process, because increased DNA methylation has not been detected at the CTCF binding site in the *FXN* 5’ UTR region of FRDA cells or tissues [15,16,25], and the increased DNA methylation at the *FXN* upstream GAA repeat region has been considered to be an unconnected marker of the heterochromatin state. However, if there is a demethylation process occurring at the *FXN* upstream GAA repeat region, as indicated by the high levels of 5hmC that we have now identified, then this may indeed be involved in increasing *FAST-1* expression, CTCF depletion and hence *FXN* gene silencing. In conclusion, we can propose a model for FRDA that combines both the increased 5hmC modification of DNA and decreased CTCF binding at the *FXN* locus. Further studies will now be required to determine the exact relationship between these molecular mechanisms and FRDA pathogenesis.
